# Nutritional Optimization for Brain Health in Contact Sports: A Systematic Review and Meta-Analysis on Long-Chain ω-3 Fatty Acids and Neurofilament Light

**DOI:** 10.1016/j.cdnut.2024.104454

**Published:** 2024-09-03

**Authors:** Jeffery L Heileson, Michael J Macartney, Nora L Watson, Tina E Sergi, Andrew R Jagim, Ryan Anthony, Gregory E Peoples

**Affiliations:** 1 Walter Reed National Medical Center, Bethesda, MD, United States; 2 Department of Health, Human Performance, and Recreation, Baylor University, Waco, TX, United States; 3 Graduate School of Medicine, University of Wollongong, Wollongong, NSW, Australia; 4 Department of Exercise and Sport Science, University of Wisconsin-La Crosse, La Crosse, WI, United States; 5 Department of Sports Medicine, Mayo Clinic Health System, La Crosse, WI, United States

**Keywords:** sports nutrition, repetitive subconcussive head impact, supplementation

## Abstract

**Background:**

Accumulating evidence has highlighted the acute and chronic impact of repetitive subconcussive head impacts (rSHIs) in contact sports. Neurofilament-light (Nf-L), a brain-derived biomarker of neuroaxonal injury, elevates in concert with rSHI. Recently, long-chain ω-3 polyunsaturated fatty acids (LC ω-3 PUFAs) supplementation has been suggested to mitigate brain injury from rSHI as reflected by attenuation of Nf-L concentrations within contact sport athletes.

**Objective:**

Using a systematic review with a meta-analysis, we aimed to determine the effect of LC ω-3 PUFA supplementation on Nf-L concentrations in athletes routinely exposed to rSHI.

**Methods:**

Electronic databases (PubMed and CINAHL) were searched from inception through January 2024. One-stage meta-analysis of individual participant-level data was used to detect changes in Nf-L concentrations between LC ω-3 PUFA and control/placebo (PL) groups from baseline to midseason (MS) and postseason (PS). Least square means (±SE) for Nf-L change from baseline were compared by treatment group for MS/PS using contrast t tests. Significance was set a priori at adjusted P ≤ 0.05.

**Results:**

Of 460 records identified, 3 studies in collegiate American football players (n = 179; LC ω-3 PUFA = 105, PL = 71) were included in the meta-analysis. Compared with PL, the change in Nf-L concentrations was statistically similar at MS [mean difference (MD) = –1.66 ± 0.82 pg·mL–1, adjusted P = 0.09] and significantly lower at PS (MD = –2.23 ± 0.83 pg·mL–1, adjusted P = 0.02) in athletes following LC ω-3 PUFA supplementation.

**Conclusions:**

Our findings demonstrate preliminary support for the prophylactic administration of LC ω-3 PUFA in contact sport athletes exposed to rSHI; however, further research is required to determine the effective dosage required.

This trial was registered at OSF (DOI: https://doi.org/10.17605/OSF.IO/EY5QW).

## Introduction

Sports-related concussion (SRC) is a type of traumatic brain injury that occurs due to the biomechanical forces from physical contact that may have both acute and chronic health consequences [1]. Despite recent advancements in protective equipment and athlete safety-focused policy and rule changes, the incidence of SRC has continued to rise [[1], [2], [3]], particularly among younger populations [4]. A potentially more insidious, yet underappreciated, concern associated with contact sports is the effects associated with repetitive subconcussive head impacts (rSHIs). Athletes participating in contact and collision sports sustain hundreds of subconcussive head impacts per season that may have long-term effects on the brain [[5], [6], [7]]. Because rSHI are asymptomatic by nature, athletes may not receive any treatment or on-field play restrictions, likely compounding the neurologic damage that can occur with repeat exposure. It is currently unknown if there is a tolerance limit to rSHI; however, brain-derived blood biomarkers have been shown to elevate in concert with head impacts, with greater increases observed for starters or players exposed to a greater number of rSHI [[8], [9], [10], [11], [12]]. As a result, these biomarkers may be used to quantify the severity of microstructural injury and track subsequent recovery over time.

One such marker, neurofilament-light (Nf-L), is considered one of the most sensitive and specific markers of detecting neuroaxonal injury following rSHI in sports [[12], [13], [14]]. Nf-L is 1 of 5 specific subtypes of neurofilaments [15] that are classified as structural proteins primarily found in neurons and, therefore, can serve as a surrogate indicator of neuronal damage [13]. Nf-L is most abundant in the deeper brain regions, specifically in the large-caliber myelinated axons. Following injury, Nf-L is released into the cerebrospinal fluid (CSF) as a result of an inflammatory cascade [12] and eventually will enter peripheral circulation, where it can be present in both serum and plasma [15]. Nf-L concentrations in blood are strongly correlated with those found in CSF, albeit in lower concentrations [15]. To date, there is no consensus on the threshold of Nf-L that would indicate clinically relevant neuronal damage or clear guidelines on what normal ranges should be. However, there is a clear relationship between the degree of neuronal damage and the severity of neurologic diseases and subsequent elevations in Nf-L [12,13,15].

Regardless of the type of contact or collision sport, there appears to be a relationship between Nf-L concentrations and the frequency and magnitude of head impacts [13,14]. In a study using an accelerometer-embedded mouthguard, the frequency and magnitude of head impacts were significantly associated with increased Nf-L concentrations in American football players [16]. Other studies have reported similar elevations in Nf-L in various sporting contexts [[8], [9], [10],[17], [18], [19]]. A study in Division II soccer players reported that serum Nf-L was significantly higher compared with active controls (12.8 pg·mL^–1^ compared with 5.7 pg·mL^–1^; *P* = 0.023) [19]. Furthermore, a recent meta-analysis of 11 studies determined that the highest concentrations of serum or CSF Nf-L were in athletes exposed to head impacts (e.g., boxing and American football) and was significantly associated with concussions and symptom severity [14].

Although much of the focus has been on SRC diagnosis and treatment postinjury, there is an emerging hypothesis regarding potential nutritional strategies in a prophylaxis role that may prevent or reduce the deleterious effects associated with SRC and rSHI**.** In 2017, Trojian et al. [20] wrote an eloquent yet controversial review article on the potential application of nutritional supplementation to treat and prevent SRC [[20], [21], [22]]. Since then, multiple reviews have acknowledged the utility of prophylactic nutritional strategies, primarily long-chain ω-3 PUFAs (LC ω-3 PUFA), in the context of rSHI and associated brain-derived biomarkers [21,[23], [24], [25]]. **A recent systematic review suggested that supplementation with LC** ω-3 PUFA (EPA and DHA) may be the most effective and safest strategy to mitigate the magnitude of brain injury in sports [24]. In fact, LC ω-3 PUFA have been referred to colloquially as “nutritional armor,” specifically regarding its roles in cognitive protection and recovery [26]. Due to their physiological functions associated with membrane phospholipid incorporation within various tissues, preconditioning may be a more appropriate description of the protective mechanisms associated with supplementation of LC ω-3 PUFA following tissue membrane incorporation. The concept of nutritional preconditioning has been used in cardiac physiology to refer to the capability of certain nutrients (i.e., LC ω-3 PUFAs) to elicit cardioprotection similar to that of ischemic or pharmacologic preconditioning [27,28]. Although preconditioning has not been applied as extensively to the brain, experimental and clinical evidence suggests that the nutritional incorporation of LC ω-3 PUFA bolsters endogenous mechanisms for protection against mechanical insults, such as rSHI [[29], [30], [31]].

Coincidently, athletes tend to exhibit low to moderate concentrations of LC ω-3 PUFA as measured via blood draw or fingerstick, which is typically expressed as the more familiar Omega-3 index (O3i, erythrocyte %DHA + %EPA). For instance, studies in athletes consistently report O3i values <5% [[32], [33], [34], [35]]. Although it is unknown if there is an ideal O3i target to optimize brain health, higher DHA and EPA concentrations have been moderately correlated with lower serum Nf-L [36]. A recent dose–response study in American football athletes suggests that DHA supplementation can increase the O3i to >8% within a season; however, the minimum length of time required was 8 wk in the 6 g·d^–1^ group, 15 wk in the 4 g·d^–1^ group, and ∼22 wk in the 2 g·d^–1^ group [37]. Although the dose may need to be higher in athletes, the O3i can be readily increased via LC ω-3 PUFA intake (dietary or supplementation) [[38], [39], [40]]. Regardless, whether a higher O3i confers any protection against the deleterious effects of rSHI remains to be determined.

Despite the potential application of LC ω-3 PUFA supplementation and preliminary data discussed, to our knowledge, no published meta-analysis has investigated the relationship of LC ω-3 PUFA on brain-derived blood biomarkers. As such, the purpose of this systematic review and meta-analysis was to determine the effect of LC ω-3 PUFA supplementation on Nf-L concentrations in athletes routinely exposed to repetitive head impacts. Based on the available literature, we aimed to identify directions for further research to advance our understanding of Nf-L, LC ω-3 PUFA, and their interaction in the context of contact sport rSHI.

## Methods

This systematic review and meta-analysis were conducted in accordance with the PRISMA guidelines. The PRISMA checklist can be viewed online in the supplementary materials (Supplemental Table 1). Our protocol was registered retrospectively with open-source framework and can be viewed at https://doi.org/10.17605/OSF.IO/EY5QW.

### Search strategy

**FIGURE 1:**
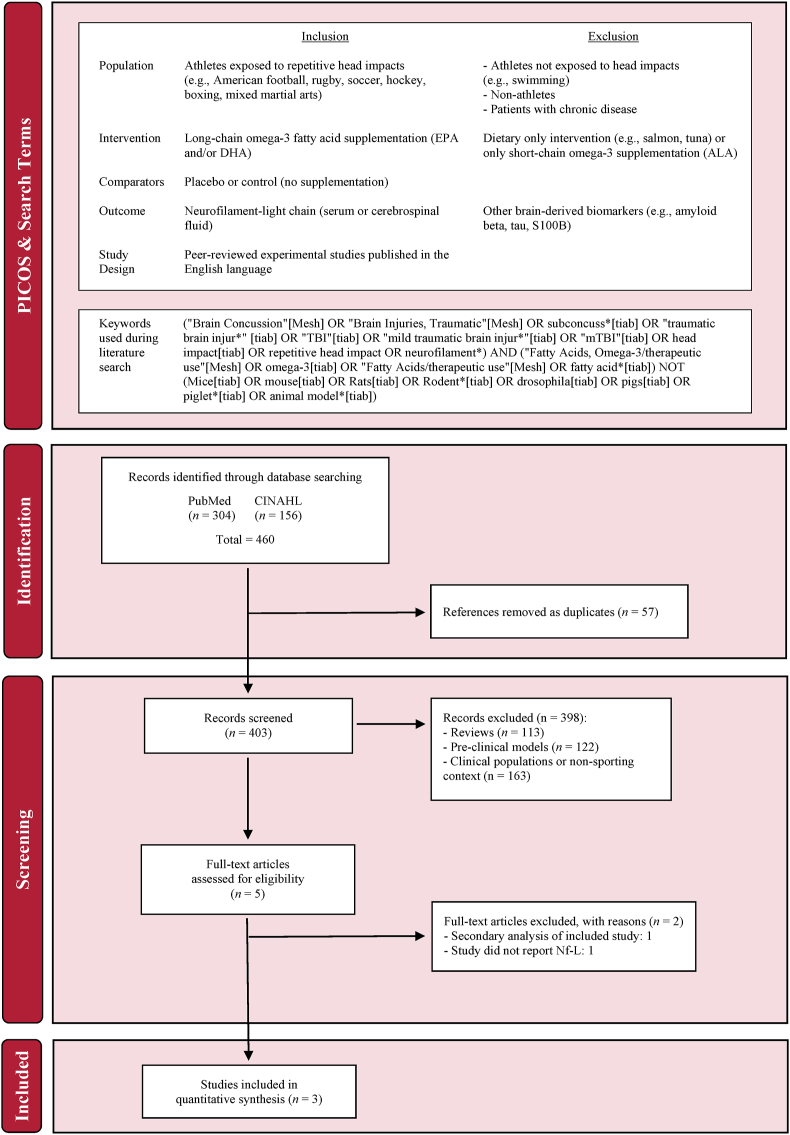
Participant, intervention, comparison, outcome, study design (PICOS) criteria, search terms, and PRISMA flow diagram.

The PubMed and CINAHL electronic databases were searched from inception until January 2024. As indicated in Figure 1, select keywords, MeSH, and matching synonyms were combined with appropriate Boolean operators to capture the most relevant literature on the specified topic.

### Study selection

Once the articles were identified and duplicates were removed, 2 authors independently screened titles and abstracts for inclusion. The Population, Intervention, Comparison, Outcome, and Study Design framework was employed to determine eligibility criteria (Figure 1). A manual search of reference lists from primary and secondary research articles failed to yield any additional studies.

### Data extraction and quality assessment

Data were extracted and entered into a custom excel spreadsheet independently by 2 authors (JLH and TES). Collected data included author/year, sample characteristics (e.g., population, sample size, and sport), LC ω-3 PUFA dose, duration of the study, Nf-L timepoints tested, LC ω-3 PUFA tissue measure, and main Nf-L outcomes.

The Cochrane Collaboration’s Risk of Bias tools were used to assess the internal validity for each randomized and nonrandom-ized trial (RoB 2 and ROBINS-I, respectively) [41,42]. For RoB 2.0, each study was classified as having either low risk, some concerns, or high risk for each domain. For ROBINS-I, each study was classified as having either low risk, moderate risk, serious risk, or critical risk for each of 7 domains. One author (JLH) judged the risk of bias, and another author verified the results (TES).

To assess the study design for LC ω-3 PUFA interventions, a 5-point quality assessment scale developed by Anthony et al. [43,44] was used. The criteria were based upon well-established best practice study design considerations for interrogating the effects of LC ω-3 PUFA in research settings [45,46]. Criteria included the following: *1*) exclusion of participants with a baseline erythrocyte membrane EPA and/or DHA concentration above a certain threshold, *2*) whether supplementation resulted in a change in erythrocyte membrane EPA and/or DHA, *3*) exclusion of participants that consumed LC ω-3 PUFA supplements, *4*) exclusion of participants consuming more than one fish meal per week, and *5*) have a minimum supplementation duration of 4 wk. The scale scored each criterion as either satisfied (1 point) or not satisfied (0 points), resulting in a score ranging from 0 to 5. Criterion *2*) was considered satisfied if the authors articulated a membrane-driven hypothesis for the tissue used to measure LC ω-3 PUFA status (e.g., plasma, whole blood compared with erythrocyte). Additionally, traffic-light labeling was used to further define studies as low-quality (red, 0–1), moderate-quality (yellow, 2–3), or high-quality (green, 4–5).

### Statistical analysis

Data analysis was performed by 1 author (NLW). Individual participant data were provided by study authors upon request. One-stage individual participant data meta-analysis used mixed models for repeated measures to compare changes in Nf-L from baseline in the treatment compared with control group at the midseason (MS) and postseason (PS) time points. The MS and PS timepoints were determined within each study. The models included fixed effects for treatment, baseline Nf-L, time point, and treatment-by-time point interaction. A random intercept was specified for subjects within the study. Fixed study-specific intercepts were estimated to allow heterogenous study effects while avoiding imprecise estimation of this variance due to the small number of studies. Restricted maximum likelihood estimation was used with the Kenward–Roger degrees of freedom method. Least square means for Nf-L change from baseline were compared by treatment group for each time point using contrast *t* tests, with *P* values corrected for comparisons at 2 time points using Bonferroni adjustment. Mixed model analyses were performed in R using the lme4 package [47], with contrasts and their *P* values generated with emmeans [48]. The thresholds for the magnitude of effects (estimated group mean difference relative to the pooled standard deviation) were classified as <0.2, trivial; 0.2, small; 0.5, medium; and 0.8, large [49]. The significance level was set a priori at adjusted *P* ≤ 0.05 for all analyses.

## Results

### Study selection

Figure 1 shows the literature search and study selection. The initial search retrieved 460 articles, which was reduced to 403 after removal of duplicates. Title and abstract screening removed all but 5 articles. Two articles were removed for not meeting predefined inclusion criteria. All contact sports were included during the search; however, only studies of American football players met the inclusion criteria. Therefore, 3 papers were included in the final meta-analysis.

### Quality assessment

**FIGURE 2:**
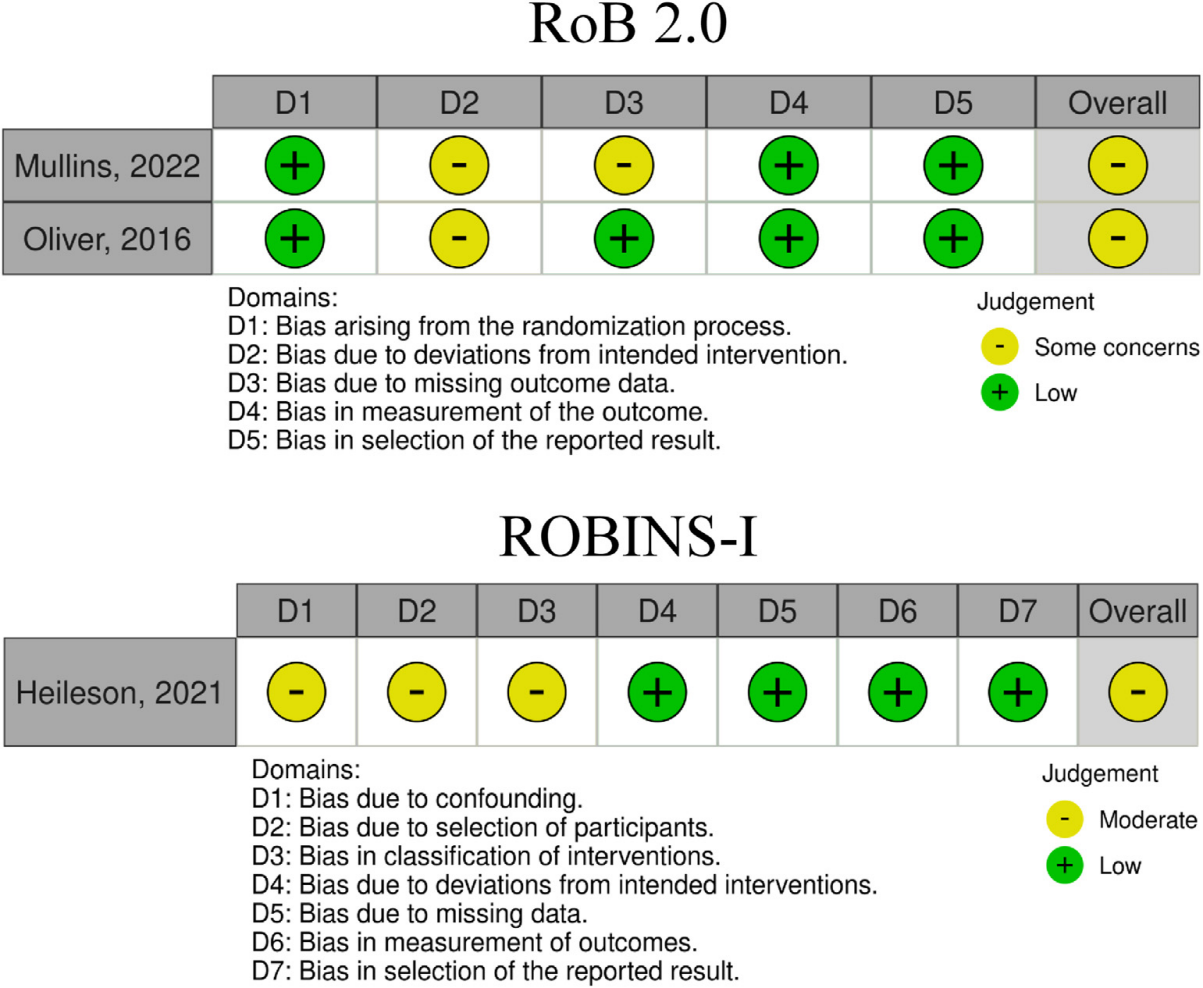
Risk of bias summary for individual trials.

All studies included had “some concerns” [36,50] or “moderate” [51] risk of bias. Study quality results based on the tool used and domains are reported in Figure 2.

The mean LC ω-3 PUFA study design score across 3 studies was 3.3 of 5 (Table 1). No study excluded participants with a baseline DHA or EPA membrane status above a set threshold (e.g., >4%). Similarly, none of the studies measured erythrocyte LC ω-3 PUFA status. However, since Oliver et al. [50] provided a justification for plasma LC ω-3 PUFA status, we deemed this as satisfying the criteria. In contrast, the remaining studies did not provide a sound reasoning for the use of plasma LC ω-3 PUFA status [36,51]. Additionally, biobanked samples from Lust et al. [37] and Oliver et al. [50] were recently analyzed for erythrocyte LC ω-3 PUFA status. All studies provided LC ω-3 PUFA supplementation (>2 g·d^–1^) for >4 wk and had inclusion/exclusion criteria based on dietary LC ω-3 PUFA intake and supplementation.

**TABLE 1:** Summary of studies investigating the effect of LC ω-3 PUFA supplementation on neurofilament-light chain (Nf-L) in American football players.

First author (y)	Participants	Dose	Duration and Nf-L timepoints (T)	LC ω-3 PUFA tissue measure	Main Nf-L outcomes	LC ω-3 PUFA score	Notes
Mullins et al. [36]	Total: *n* = 29 PL: *n* = 17 ω-3: *n* = 12	Total: 3.5 g·d^–1^ EPA: 1.0 g DHA: 2.4 g	33 wk T1: Baseline T2: Midseason T3: Postseason From T1-T3 was 26 wk	Plasma ω-3 ↑ EPA ↑ DHA ↑ EPA was similar to PL at T3	• Nf-L increased similarly in both groups • ω-3 did not attenuate Nf-L concentrations		• Dropout rate was higher in the ω-3 group (37% vs. 11%) • Underpowered • Plasma DHA and EPA peaked at 8 wk • Plasma DHA + EPA concentrations were negatively associated with Nf-L at T2 (*r* = –0.42) and T3 (*r* = –0.44). • Upset stomach reported (*n* = 3)
Heileson et al. [51]	Total: *n* = 66 PL: *n* = 35 ω-3: *n* = 31	Total: 3.0 g·d^–1^ EPA: 0.6 g DHA: 2.0 g	∼20 wk T1: Baseline T2: After preseason T3: ∼10 wk T4: Midseason T5: ∼16 wk T6: Postseason	Plasma ω-3 ↑ EPA ↑ DHA ↑	• Nf-L was only elevated in the control team • Nf-L was significantly lower than PL at T2, T3, and T4		• Nonrandomized trial • Compared athletes from different concentrations of competition (DI vs. DIII) • Athletes with <80% supplement compliance were excluded from analysis
Oliver et al. [50]	Total: *n* = 81 PL: *n* = 19 ω-3: *n* = 62	Three DHA groups: • 2.0 g • 4.0 g • 6.0 g	27 wk T1: Baseline T2: Before preseason T3: After preseason T4: ∼11 wk T5: ∼15 wk T6: Midseason T7: ∼23 wk T8: Postseason	Plasma ω-3 ↑ EPA ↔ DHA ↑ EPA was only increased in the 6.0 g group	• When ω-3 groups were combined, Nf-L was significantly lower at T7 and T8 • When analyzed by groups, only the 2 g·d^–1^ DHA group substantially decreased Nf-L. • At T8, 4 g·d^–1^ DHA likely attenuated Nf-L; whereas 6 g·d^–1^ DHA had an unclear effect on Nf-L		• Athletes with <80% supplement compliance were excluded from analysis • GI distress reported (*n* = 4)

Abbreviations: LC ω-3 PUFA, long-chain ω-3 polyunsaturated fatty acids; Nf-L, neurofilament-light; PL, placebo.

↑, significant increase; ↓, significant decrease; ↔, no significant difference; GI, gastrointestinal.

LC ω-3 PUFA Study Design Score adapted from Anthony et al. [44], indicates trial quality, red = low (0–1), yellow = moderate (2–3), and green = high (4–5).

## Results of Individual Studies

Characteristics and details of the 3 included studies are summarized in Table 1 [36,50,51]. Every study was conducted throughout a collegiate American football season ranging between 20 and 33wk and included between 29 and 81 athletes. The range of LC ω-3 PUFA dose varied across studies from 2 to 6 g·d^–1^ and contained DHA only or a mixture of LC ω-3 PUFA (EPA and DHA). All studies reported baseline DHA + EPA <5% and significant increases in plasma LC ω-3 PUFA status following supplementation. Two studies reported plasma LC ω-3 PUFA status at preseason and PS [50,51], whereas Mullins et al. [36] presented multiple timepoints. Notably, erythrocyte fatty-acid status was recently reported at multiple timepoints from biobanked samples from Oliver et al. [37,50]. From preseason to PS, DHA + EPA increased by ∼53%, 71.6%, and ∼105% in Mullins et al. [36], Heileson et al. [51], and Oliver et al. [50], respectively. Two studies were RCTs [36,50] and 1 study was a nonrandomized trial with a control group [51]. Oliver et al. [50] explored the effect of varying doses of DHA (2, 4, and 6 g·d^–1^) compared with placebo (PL) on Nf-L throughout a 27-wk season (T1–T8). Due to the lack of change in serum Nf-L in nonstarters, data were only analyzed in athletes identified as starters (ie., players with the greatest exposure to rSHI). More specifically, starters were defined as the athletes known to go out with the first or second team, first or second on the depth roster, and take a majority of the repetitions (∼20–40+ per game).

Regardless of dose (i.e., collapsed across treatments), DHA supplementation in a study by Oliver et al. [50] trended toward attenuation of Nf-L after rigorous preseason training (T3, *P* = 0.07) and significantly attenuated Nf-L elevations near and at the end of the regular season (T7, *P* = 0.022 and T8, *P* = 0.012). Interestingly, the greatest reduction in Nf-L compared with PL was observed in the 2 g·d^–1^ group. Unfortunately, the number of head impacts was not collected; hence, it is difficult to ascertain if this relationship was due to differential stress stimulus, rather than the dose. Most importantly, the low number of athletes per group (e.g., 3 players in the 6 g·d^–1^ group) limits statistical inferences, yet all groups had substantially lower Nf-L (∼60%–100%) compared with PL. Heileson et al. [51] supplemented football players with 2.56 g·d^–1^ LC ω-3 PUFA (2 g DHA, 0.56 g EPA) and examined changes in Nf-L over the season (T1–T6). However, this study was nonrandomized and utilized a control team, in a less competitive Division (DIII compared with DI), to compare Nf-L concentrations rather than using a PL. Supplementation with LC ω-3 PUFA significantly attenuated Nf-L throughout the season compared with the control group (*P* = 0.024). When analyzing starters only, Nf-L was not significantly elevated in the LC ω-3 PUFA group, whereas those in the control group experienced significant increases (*P* < 0.05) throughout the season. However, there were no group-by-treatment effects noted. In contrast to the previous studies, the most recent investigation by Mullins et al. [36] supplemented football players with 3.5 g·d^–1^ LC ω-3 PUFAs (2.4 g DHA and 1.0 g EPA) or a PL for 26 wk; however, Nf-L was similar between groups throughout the season [36]. Interestingly, when dichotomized as starters and nonstarters, Nf-L was only significantly elevated in starters compared with baseline (T2, *P* = 0.012 and T3, *P* < 0.001) with no significant increases in nonstarters. However, Mullins et al. [36] had the smallest sample size (*n* = 29: ω–3, *n* = 12; PL, *n* = 17) and, based on the plasma fatty-acid data, may have suffered from compliance issues.

### Meta-analysis

**FIGURE 3:**
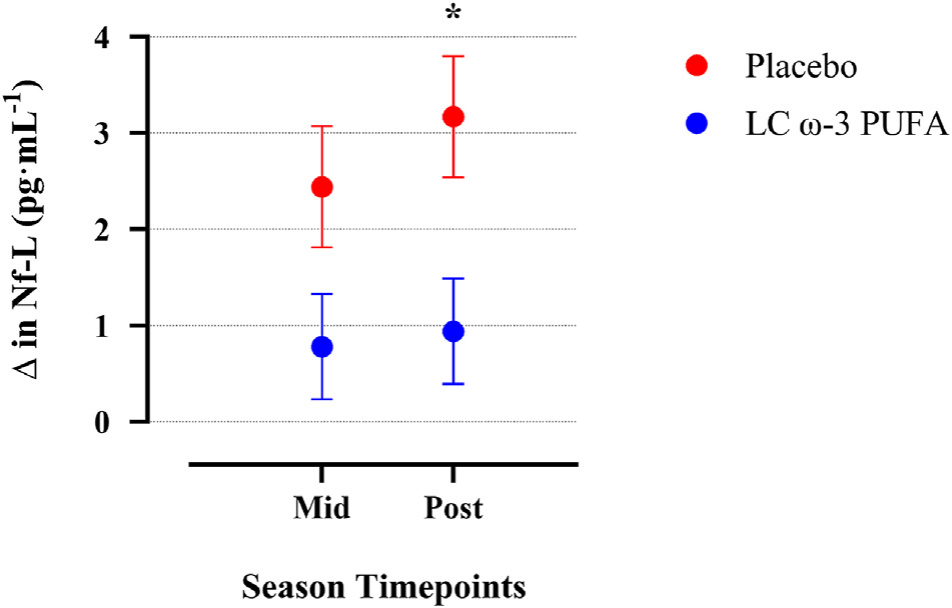
Individual participant data meta-analysis on the effect of long-chain ω-3 PUFAs (LC ω-3 PUFA, *n* = 105) on the change in neurofilament-light (Nf-L, mean difference ± SE) compared with placebo (*n* = 71) in American football players at midseason (mid) and postseason (post). In the aggregate of LC ω-3 PUFA groups, Nf-L was nonsignificantly attenuated at mid (adjusted *P* = 0.09) and significantly attenuated at post (∗adjusted *P* = 0.02). Nf-L, neurofilament-light.

The meta-analysis comprised aggregating the individual participant-level data (*n* = 179; LC ω-3 PUFA = 105, PL = 71) at baseline, MS, and PS. The MS and PS timepoints were determined within each study. Mullins et al. [36] only reported baseline, MS and PS; whereas MS/PS was T6/T8 and T4/T6 for Oliver et al. [50] and Heileson et al. [51], respectively (Table 1). Additionally, Oliver et al. [50] combined 3 treatment groups (2, 4, and 6 g) into 1 group for analysis. The groups were combined to avoid a unit-of-analysis error, as recommended by Cochrane [52]. In mixed model analyses of LC ω-3 PUFA on Nf-L change, Nf-L increases from baseline were lower in the ω-3 group compared with the PL groups at both timepoints analyzed (Figure 3). After correcting for comparisons at 2 time points, Nf-L change at MS was not statistically different between conditions [least square mean difference = –1.66 pg·mL^–1^ (SE = 0.82); adjusted *P* = 0.09; Hedge’s *g* = 0.40]. At PS, Nf-L was significantly attenuated in the LC ω-3 PUFA group compared with PL [least square mean difference = –2.23 pg·mL^–1^ (SE = 0.83); adjusted *P* = 0.02; Hedge’s *g* = 0.37].

## Discussion

This systematic review examined the effect of LC ω-3 PUFA supplementation on Nf-L in contact sport athletes exposed to rSHI. Although the evidence is limited, the data presented herein indicate that LC ω-3 PUFA supplementation attenuates the elevation of Nf-L throughout a collegiate football season, primarily in starters; however, the effect is regarded as *small*. Interestingly, the between group mean difference in serum Nf-L concentrations increased with the length of supplementation, which highlights the potential importance of LC ω-3 PUFA optimization, through membrane incorporation, for brain health and its neuroprotective benefits. Despite the findings, we consider the evidence preliminary rather than conclusive, and therefore more hypothesis-driven and carefully designed research is warranted.

Nonetheless, our findings also highlight the importance of optimal LC ω-3 PUFA concentrations, especially because athletes tend to consume poor dietary sources of LC ω-3 PUFA and have low baseline O3i status [[32], [33], [34], [35]]. Although it is recommended that athletes consume 2 g·d^–1^ LC ω-3 PUFA [53,54], the current evidence suggests that athletes tend to consume <500 mg·d^–1^, even when taking LC ω-3 PUFA supplements [32,34,[55], [56], [57], [58]]. As expected, recent studies have reported that athletes tend to have an O3i between 4.0% and 4.6% [[32], [33], [34], [35],^57^,^58^], indicating that there may be insufficient LC ω-3 PUFA available for tissue membrane incorporation (i.e., submaximal preconditioning of DHA and/or EPA within the target tissues). In conjunction, certain nondietary determinants may influence LC ω-3 PUFA status in athletes, such as sex, body mass, and rigorous physical training environments [[59], [60], [61], [62]]. However, a dose–response trial determined that 68% of the change in the O3i was explained by the dose, whereas the combination of body weight, physical activity, and sex only explained ∼10% of the variability in response [59]. More recently, longitudinal studies have consistently reported that athletes, male and female, experience an immediate and sustained reduction in blood LC ω-3 PUFA status, ranging between 0.9% and 2.0%, following 12–28 d of training, including competition and multisession days [61,62]. Because diet intake was not controlled or accounted for, the depletion of fatty-acid status may not be due to physical activity, but a change in dietary patterns, such as greater carbohydrate and protein intake at the expense of LC ω-3 PUFAs. In agreement with Flock et al. [59], a recent study on military trainees suggests that the decline in the O3i throughout training is most likely attributed to a Westernized diet, not the training environment *per se* [63]. In fact, Macartney et al. [38] determined that O3i status can be maintained during physiologically demanding cycling with consistent intake of 0.75–1.5 g·d^–1^ LC ω-3 PUFAs (1–2 capsules). As such, LC ω-3 PUFA status can be readily manipulated by the dietary intake of fatty fish or low-cost supplementation [64], even in athletes subjected to intense training regimes [[38], [39], [40]]. Of course, maximal or near maximal LC ω-3 PUFA incorporation may take 2–4 mo, depending primarily on the dose provided [37]. In the United States, the National Collegiate Athletic Association recently deemed LC ω-3 PUFA supplementation a “permissible” nutritional supplement for student-athletes [65].

Two of the 3 studies reported consistent evidence that Nf-L, a serum biomarker of neuroaxonal injury, is not elevated in the presence of LC ω-3 PUFA supplementation; however, the most recent investigation suggests that LC ω-3 PUFA intake does not influence Nf-L. The conflicting results from Mullins et al. [36] may be attributable to supplement noncompliance combined with unremarkable changes in Nf-L concentrations. Furthermore, the dropout rate was also higher in the LC ω-3 PUFA supplementation group than the PL group. Plasma DHA and EPA peaked at 8 wk and remained relatively stable through MS. However, DHA and EPA precipitously declined from MS until PS. Supplement compliance can be easily measured by converting plasma DHA + EPA into the O3i at baseline and PS [66]. Based on data from Mullins et al. [36] (Supplemental Table S2), absolute O3i was only ∼1.15% higher than baseline. Using validated equations by Walker et al. [67], the ethyl ester dose provided would conservatively predict an O3i increase by ≥3.5%. Over a similar timeframe, erythrocyte data from Oliver et al. [37,50] determined that just 2 g·d^–1^ DHA increased the O3i by ∼3%. For all conditions, erythrocyte DHA remained 2–3× higher than PL throughout the study. Similarly, Heileson et al. [51] provided athletes with less DHA (∼0.4 g·d^–1^) and EPA (∼0.4 g·d^–1^); despite this, the relative response to treatment was ∼45% and 16% higher for plasma DHA and EPA, respectively, than Mullins et al. [36]. In fact, this equates to a similar average O3i increase (∼3%), as reported by Lust et al. [37]. When the O3i was nearly at its peak (MS), Nf-L did not significantly increase in the LC ω-3 PUFA group and was lower than PL (*d* = 0.31), but as supplementation compliance likely declined, Nf-L was at its highest. In this context, the nutritional optimization of LC ω-3 PUFA hypothesis per se was not tested, limiting any inference from the study’s outcomes.

Of particular interest, Nf-L concentrations in Mullins et al. [36] were substantially lower at MS (∼10%–30%) and PS (∼20%–90%) than in previous investigations [50,51]. The nominal range of Nf-L values, 2.3–15.5 pg·mL^–1^, most likely limited the ability to detect any potential effect of LC ω-3 PUFA supplementation. Notably, the peak Nf-L value was from a single player, whereas all other values were <12 pg·mL^–1^. Other moderating factors, such as suboptimal sample size and stratification decisions (lineman as high risk, speed players as low risk [e.g., wide receiver]) could have contributed to the divergent findings [17]. Nonetheless, Mullins et al. [36] reported that %DHA + %EPA were moderately correlated with Nf-L concentrations at MS (*r* = –0.42, *P* = 0.17) and PS (*r* = –0.44, *P* = 0.15). Furthermore, a subset of athletes had additional neuroimaging to investigate the potential neuroprotective effects of LC ω-3 PUFA supplementation [68]. Although DHA and EPA did not prevent white matter damage caused by rSHI, supplementation with LC ω-3 PUFA preserved the brain’s structural and cross-functional connectivity. This provides some credence to the hypothesis that higher concentrations of DHA and EPA (i.e., optimization) may attenuate axonal injury, as reflected by measurement of Nf-L concentrations, and is more consistent to previous studies upon critical evaluation.

This review builds upon an emerging nutritional frontier in supporting the brain health of athletes at risk of rSHI. The incorporation of EPA and DHA into cell membranes for lowering cardiovascular disease risk has been well-established and are now complemented by the emerging potential of LC ω-3 PUFA for neural protection, marking a significant advancement in support of this hypothesis. At this pivotal stage, there is an urgency for carefully designed and appropriate studies that include “stringent controls” and objective outcomes specific to the hypothesis being examined [69]. Unfortunately, these shortcomings are exemplified by studies that have attempted to examine the influence of EPA and DHA in trained or athletic populations [44] which can lead to confusion about the physiological roles of both fatty acids. For some time, there has been discussion regarding the “balance” of these 2 LC ω-3 PUFAs, although in terms of the O3i, DHA is regarded to be the major contributor to optimization [70]. In terms of athlete nutrition, the wholistic approach of total diet ω-6/ω-3 has also been discussed at length, in terms of supporting physical training [71]. Although it is commendable to reduce the total ω-6 PUFA in the diet, especially in the context of a Western-style diet, the improvement to physiological health is likely multifactorial and primarily the research surrounding this ω-6/ω-3 ratio has focused primarily on chronic cardiometabolic health [72] and inflammatory-based pathologies [73]. To our knowledge, there have been no hypothesis-driven studies manipulating, specifically, the intake of ω-6 and ω-3 (and especially EPA and DHA) in trained populations. Rather, where there has been varied EPA and DHA supplemental intake, it has more likely been explained by the *ad hoc* provision of various commercially available ω-3 supplements, which vary extensively depending upon the marine source [44]. Nevertheless, despite two-thirds of these studies in physically trained populations using a ω-3 supplement predominating with EPA [43,44], this only reflects the plasma concentrations over the acute time period and not ultimately the membrane phospholipids [74]. In fact, the principle of membrane incorporation, favoring DHA for many tissues such as the heart, occurs independently of the background ω-6 provision or competition with EPA [75]. On first principles, decisions on dose and duration of EPA and/or DHA intake should be underscored by reasoning that includes whether the potential benefit is gained through circulating concentrations or the critical step of membrane incorporation. For the latter, this will mean treating EPA and DHA as unique fatty acids, given their differences in membrane incorporation, best exemplified by cardiac and skeletal muscle where DHA is the predominant phospholipid [76]. For neural cells, there seems to be a similar preference for DHA for physiological optimization [77] although the challenge for primary outcomes relating to concussion is further understanding the time course for membrane incorporation specific to the physiological stress of rSHI.

Notwithstanding, this current analysis must also recognize the limited scope of its findings, at this early stage. First, our analysis only included 3 studies, which heightens the impact of a single divergent timepoint and our uncertainty of the evidence. Nonetheless, given the rapid development of concussion-related outcomes for contact athletes, we feel that early aggregated evidence, as presented in this review, is important to adjust study designs to establish or reject the potential role of LC ω -3 PUFA for supporting athletes exposed to rSHI. Second, the dose and type of LC ω-3 PUFA supplements provided were inconsistent across interventions. All studies provided ≥2 g·d^–1^ DHA with positive results noted with no or proportionally lower EPA. Third, contact data from helmets would allow more insight into the dose response of contacts and change in Nf-L. Lastly, Nf-L has been described as a potential diagnostic and prognostic biomarker of rSHI; however, to date, there are no defined clinical cutoffs, meaning the outcomes are limited to a physiological description.

In conclusion, for the first time, we have evaluated the aggregate effect of LC ω-3 PUFA supplementation on Nf-L in American football players. The findings demonstrate initial empirical support for the prophylactic administration of LC ω-3 PUFA, containing ≥2 g·d^–1^ DHA, in contact sport athletes typically exposed to repetitive head impacts. However, given that athletes tend to consume <500 mg·d^–1^ through their habitual diet, further research is necessary to determine whether doses <2 g·d^–1^ DHA are also effective and this will rely on emerging evidence about how the brain incorporates LC ω-3 PUFA into its cell membranes for the primary function of maintaining DHA as part of the phospholipids during adulthood. Although our analysis only included American football players, athletes from other contact sports (e.g., boxing, mixed martial arts, and rugby) may equally benefit from nutritional optimization via LC ω-3 PUFA intake. The preliminary findings presented herein, although positive, need to be replicated in various sporting contexts (e.g., rugby), and when possible, record the number of head impacts athletes are exposed to. Additionally, future studies should explore the potential differential effects (or noneffects) of EPA and DHA on brain-derived biomarkers while adhering to the well-established LC ω-3 PUFA intervention best practices. Furthermore, information regarding changes in O3i can provide additional insight into compliance, effectiveness of the supplementation regiment and relationships between O3i and severity of injury or timeline of recovery. Collectively, our findings point toward the need for more well-designed research to build upon the evidence on the relationship between preformed LC ω-3 PUFA and brain-derived biomarkers. Subsequent trials can help inform sport nutrition guidelines for daily DHA and EPA intake recommendations (food and supplement) to support a wide range of athlete health and fitness markers, particularly for contact sport athletes who experience extensive physiological strain to multiple body systems, exemplified by rSHI.

## Acknowledgments

The views expressed in this manuscript are those of the author(s) and do not necessarily reflect the official policy of the Department of Defense or the US Government.

## Author contributions

The authors’ responsibilities were as follows – JLH, MJM, RA, GEP: were involved in the conceptualization of the review; JLH, TES, ARJ, NLW: conducted the literature review and/or assisted with figure and table production; NLW: performed the meta-analysis; and all authors: significantly contributed to writing the manuscript and approved the final version for publication.

## Conflict of interest

The authors report no conflicts of interest.

## Funding

The authors reported no funding received for this study.

## Data availability

Data described in the manuscript, code book, and analytic code will be made available upon reasonable request.

## References

[1] Hallock H., Mantwill M., Vajkoczy P., Wolfarth B., Reinsberger C., Lampit A. (2023). Sport-related concussion: a cognitive perspective. Neurol. Clin. Pract..

[2] Eliason P.H., Galarneau J.-M., Kolstad A.T., Pankow M.P., West S.W., Bailey S. (2023). Prevention strategies and modifiable risk factors for sport-related concussions and head impacts: a systematic review and meta-analysis. Br. J. Sports Med..

[3] King D.A., Clark T.N., Hume P.A., Hind K. (2022). Match and training injury incidence in rugby league: a systematic review, pooled analysis, and update on published studies, Sports Med. Health Sci..

[4] Askow A.T., Erickson J.L., Jagim A.R. (2020). Recent trends in youth concussions: a brief report. J. Prim. Care Community Health..

[5] McKee A.C., Alosco M.L., Huber B.R. (2016). Repetitive head impacts and chronic traumatic encephalopathy. Neurosurg. Clin. N. Am..

[6] Mackay D.F., Russell E.R., Stewart K., MacLean J.A., Pell J.P., Stewart W. (2019). Neurodegenerative disease mortality among former professional soccer players. N. Engl. J. Med..

[7] Bailes J.E., Petraglia A.L., Omalu B.I., Nauman E., Talavage T. (2013). Role of subconcussion in repetitive mild traumatic brain injury. J. Neurosurg..

[8] Oliver J.M., Jones M.T., Kirk K.M., Gable D.A., Repshas J.T., Johnson T.A. (2016). Serum neurofilament light in American football athletes over the course of a season. J. Neurotrauma.

[9] Oliver J.M., Anzalone A.J., Stone J.D., Turner S.M., Blueitt D., Garrison J.C. (2019). Fluctuations in blood biomarkers of head trauma in NCAA football athletes over the course of a season. J. Neurosurg..

[10] Shahim P., Tegner Y., Marklund N., Blennow K., Zetterberg H. (2018). Neurofilament light and tau as blood biomarkers for sports-related concussion. Neurology.

[11] Wirsching A., Chen Z., Bevilacqua Z.W., Huibregtse M.E., Kawata K. (2019). Association of acute increase in plasma neurofilament light with repetitive subconcussive head impacts: a pilot randomized control trial. J. Neurotrauma.

[12] Zetterberg H., Smith D.H., Blennow K. (2013). Biomarkers of mild traumatic brain injury in cerebrospinal fluid and blood. Nat. Rev. Neurol..

[13] Verduyn C., Bjerke M., Duerinck J., Engelborghs S., Peers K., Versijpt J. (2021). CSF and blood neurofilament levels in athletes participating in physical contact sports: a systematic review. Neurology.

[14] Karantali E., Kazis D., McKenna J., Chatzikonstantinou S., Petridis F., Mavroudis I. (2022). Neurofilament light chain in patients with a concussion or head impacts: a systematic review and meta-analysis. Eur. J. Trauma Emerg. Surg..

[15] Yuan A., Nixon R.A. (2021). Neurofilament proteins as biomarkers to monitor neurological diseases and the efficacy of therapies. Front. Neurosci..

[16] Rubin L.H., Tierney R., Kawata K., Wesley L., Lee J.H., Blennow K. (2019). NFL blood levels are moderated by subconcussive impacts in a cohort of college football players. Brain Inj.

[17] Papa L., Walter A.E., Wilkes J.R., Clonts H.S., Johnson B., Slobounov S.M. (2022). Effect of player position on serum biomarkers during participation in a season of collegiate football. J. Neurotrauma.

[18] Shahim P., Zetterberg H., Tegner Y., Blennow K. (2017). Serum neurofilament light as a biomarker for mild traumatic brain injury in contact sports. Neurology.

[19] Antonio J., Cabrera D., Knafo S., Thomas J., Peacock C., Tartar J. (2021). Neurofilament Light (NFL) in Division II female soccer players: a potential biomarker for brain trauma. JEPonline.

[20] Trojian T.H., Wang D.H., Leddy J.J. (2017). Nutritional supplements for the treatment and prevention of sports-related concussion-evidence still lacking, Curr. Sports Med. Rep..

[21] Oliver J.M., Anzalone A.J., Jones M.T., Kirk K.M., Gable D.A., Gao Y. (2018). Nutritional supplements for the treatment and prevention of sports-related concussion – omega 3 fatty acids: evidence still lacking?. Curr. Sports Med. Rep..

[22] Trojian T.H., Leddy J.J., Wang D.H. (2018). Response to the letter to the editor. Curr. Sports Med. Rep..

[23] Lust C.A.C., Mountjoy M., Robinson L.E., Oliver J.M., Ma D.W.L. (2020). Sports-related concussions and subconcussive impacts in athletes: incidence, diagnosis, and the emerging role of EPA and DHA. Appl. Physiol. Nutr. Metab..

[24] Feinberg C., Dickerson Mayes K., Jarvis R.C., Carr C., Mannix R. (2023). Nutritional supplement and dietary interventions as a prophylaxis or treatment of sub-concussive repetitive head impact and mild traumatic brain injury: a systematic review. J. Neurotrauma..

[25] Tomczyk M., Heileson J.L., Babiarz M., Calder P.C. (2023). Athletes can benefit from increased intake of EPA and DHA-evaluating the evidence. Nutrients.

[26] Kim H.-Y. (2014). Neuroprotection by docosahexaenoic acid in brain injury. Mil. Med..

[27] Abdukeyum G.G., Owen A.J., McLennan P.L. (2008). Dietary (n-3) long-chain polyunsaturated fatty acids inhibit ischemia and reperfusion arrhythmias and infarction in rat heart not enhanced by ischemic preconditioning. J. Nutr..

[28] McLennan P.L. (2014). Cardiac physiology and clinical efficacy of dietary fish oil clarified through cellular mechanisms of omega-3 polyunsaturated fatty acids. Eur. J. Appl. Physiol..

[29] Barrett E.C., McBurney M.I., Ciappio E.D. (2014). ω-3 fatty acid supplementation as a potential therapeutic aid for the recovery from mild traumatic brain injury/concussion. Adv. Nutr..

[30] Wen J., Satyanarayanan S.K., Li A., Yan L., Zhao Z., Yuan Q. (2024). Unraveling the impact of omega-3 polyunsaturated fatty acids on blood–brain barrier (BBB) integrity and glymphatic function. Brain Behav. Immun..

[31] Patch C.S., Hill-Yardin E.L., Lewis M., Ryan L., Daly E., Pearce A.J. (2021). The more, the better: high-dose Omega-3 fatty acids improve behavioural and molecular outcomes in preclinical models in mild brain injury. Curr. Neurol. Neurosci. Rep..

[32] Heileson J.L., Elliott A., Buzzard J.A., Cholewinski M.C., Jackson K.H., Gallucci A. (2023). A cross-sectional analysis of whole blood long-chain ω-3 polyunsaturated fatty acids and its relationship with dietary intake, body composition, and measures of strength and power in collegiate athletes. J. Am. Nutr. Assoc..

[33] Armstrong A., Anzalone A.J., Pethick W., Murray H., Dahlquist D.T., Askow A.T. (2021). An evaluation of omega-3 status and intake in Canadian Elite Rugby 7s players. Nutrients.

[34] Ritz P.P., Rogers M.B., Zabinsky J.S., Hedrick V.E., Rockwell J.A., Rimer E.G. (2020). Dietary and biological assessment of the omega-3 status of collegiate athletes: a cross-sectional analysis. PLOS ONE.

[35] Anzalone A., Carbuhn A., Jones L., Gallop A., Smith A., Johnson P. (2019). The omega-3 index in National Collegiate Athletic Association Division I collegiate football athletes. J. Athl. Train..

[36] Mullins V.A., Graham S., Cummings D., Wood A., Ovando V., Skulas-Ray A.C. (2022). Effects of fish oil on biomarkers of axonal injury and inflammation in American Football Players: a placebo-controlled randomized controlled trial. Nutrients.

[37] Lust C.A.C., Burns J.L., Jones M.T., Smith S.B., Choi S.H., Krk M. (2023). The dose-response effect of docosahexaenoic acid on the omega-3 index in American football athletes. Med. Sci. Sports Exerc..

[38] Macartney M., Hesseling M., Ortolano R., McLennan P., Peoples G. (2021). Evaluating the effect of a fish oil supplement on the omega-3 index of three professional cyclists competing in the Tour de France: a case study: omega-3 index and professional road cycling. J. Sci. Cycling..

[39] Macartney M.J., Hesseling M., Ortolano R., McLennan P.L., Peoples G.E. (2023). Assessing the omega-3 index of a professional cycling team and the influence of ad libitum provision of fish oil during the competitive season. J. Sport Exerc. Sci..

[40] Anthony R., Jaffrey N., Byron C., Peoples G.E., Macartney M.J. (2024). Omega-3 status evaluation in Australian female rugby league athletes: ad libitum fish oil provision results in a varied omega-3 index. Int. J. Sport. Nutr. Exerc. Metab..

[41] Sterne J.A.C., Savović J., Page M.J., Elbers R.G., Blencowe N.S., Boutron I. (2019). RoB 2: a revised tool for assessing risk of bias in randomised trials. BMJ.

[42] Sterne J.A., Hernán M.A., Reeves B.C., Savović J., Berkman N.D., Viswanathan M. (2016). ROBINS-I: a tool for assessing risk of bias in non-randomised studies of interventions. BMJ.

[43] Anthony R., Macartney M.J., Peoples G.E. (2021). The influence of long-chain omega-3 fatty acids on eccentric exercise-induced delayed muscle soreness: reported outcomes are compromised by study design issues. Int. J. Sport Nutr. Exerc. Metab..

[44] Anthony R., Macartney M.J., Heileson J.L., McLennan P.L., Peoples G.E. (2024). A review and evaluation of study design considerations for omega-3 fatty acid supplementation trials in physically trained participants. Nutr. Res. Rev.

[45] James M.J., Sullivan T.R., Metcalf R.G., Cleland L.G. (2014). Pitfalls in the use of randomised controlled trials for fish oil studies with cardiac patients. Br. J. Nutr..

[46] de Groot R.H.M., Meyer B.J. (2020). ISSFAL Official Statement Number 6: the importance of measuring blood omega-3 long chain polyunsaturated fatty acid levels in research, Prostaglandins Leukot. Essent. Fatty Acids.

[47] Bates D., Mächler M., Bolker B., Walker S. (2015). Fitting linear mixed-effects models using lme4. J. Stat. Softw..

[48] Lenth R. emmeans: estimated marginal means, aka least-squares means. https://CRAN.R-project.org/package=emmeans.

[49] Cohen J. (1988).

[50] Oliver J.M., Jones M.T., Kirk K.M., Gable D.A., Repshas J.T., Johnson T.A. (2016). Effect of docosahexaenoic acid on a biomarker of head trauma in American football. Med. Sci. Sports Exerc..

[51] Heileson J.L., Anzalone A.J., Carbuhn A.F., Askow A.T., Stone J.D., Turner S.M. (2021). The effect of omega-3 fatty acids on a biomarker of head trauma in NCAA football athletes: a multi-site, non-randomized study. J. Int. Soc. Sports Nutr..

[52] Higgins JPT, Thomas J, Chandler J, Cumpston M, Li T, Page MJ, Welch VA (editors). Cochrane Handbook for Systematic Reviews of Interventions version 6.4 (August 2023). Cochrane, 2023. Available from www.training.cochrane.org/handbook.

[53] Maughan R.J., Burke L.M., Dvorak J., Larson-Meyer D.E., Peeling P., Phillips S.M. (2018). IOC consensus statement: dietary supplements and the high-performance athlete. Int. J. Sport Nutr. Exerc. Metab..

[54] Rawson E.S., Miles M.P., Larson-Meyer D.E. (2018). Dietary supplements for health, adaptation, and recovery in athletes. Int. J. Sport Nutr. Exerc. Metab..

[55] Carbuhn A.F., D’Silva L.J. (2024). Red blood cell omega-3 fatty acid content is negatively associated with purposeful gameplay header frequencies in collegiate women soccer players: implications for diet and brain health. Nutr. Health.

[56] Wilson P.B., Madrigal L.A. (2016). Associations between whole blood and dietary omega-3 polyunsaturated fatty acid levels in collegiate athletes. Int. J. Sport Nutr. Exerc. Metab..

[57] Zhang Q., Xu Q., Tian H., Chu Y., Qiu J., Sun M. (2023). Serum and diet long-chain omega-3 fatty acid nutritional status in Chinese elite athletes. Lipids.

[58] Essman M., Christifano D., Sullivan D.K., Chalise P., Carbuhn A. (2022). Assessing omega-3 intake in sport: the Brief Food Frequency Questionnaire and the omega-3 index in collegiate women soccer players. J. Athl. Train..

[59] Flock M.R., Skulas-Ray A.C., Harris W.S., Etherton T.D., Fleming J.A., Kris-Etherton P.M. (2013). Determinants of erythrocyte omega-3 fatty acid content in response to fish oil supplementation: a dose–response randomized controlled trial. J. Am. Heart Assoc..

[60] Nikolaidis M.G., Mougios V. (2004). Effects of exercise on the fatty-acid composition of blood and tissue lipids. Sports Med.

[61] Huggins R.A., Fortunati A.R., Curtis R.M., Looney D.P., West C.A., Lee E.C. (2019). Monitoring blood biomarkers and training load throughout a collegiate soccer season. J. Strength Cond. Res..

[62] Walker A.J., McFadden B.A., Sanders D.J., Rabideau M.M., Hofacker M.L., Arent S.M. (2019). Biomarker response to a competitive season in Division I female soccer players. J. Strength Cond. Res..

[63] Peoples G.E., Larsen P., Bowes H.M., Coombes J., Drain J.R., Groeller H. (2022). The influence of a basic military training diet on whole blood fatty acid profile and the omega-3 index of Australian Army recruits. Appl. Physiol. Nutr. Metab..

[64] Dempsey M., Rockwell M.S., Wentz L.M. (2023). The influence of dietary and supplemental omega-3 fatty acids on the omega-3 index: a scoping review. Front. Nutr..

[65] (2020). NCAA, Division I Manual [Internet]. http://www.ncaapublications.com/p-4605-2020-2021-ncaa-division-i-manual.aspx.

[66] Stark K.D., Aristizabal Henao J.J., Metherel A.H., Pilote L. (2016). Translating plasma and whole blood fatty acid compositional data into the sum of eicosapentaenoic and docosahexaenoic acid in erythrocytes. Prostaglandins Leukot. Essent. Fatty Acids.

[67] Walker R.E., Jackson K.H., Tintle N.L., Shearer G.C., Bernasconi A., Masson S. (2019). Predicting the effects of supplemental EPA and DHA on the omega-3 index. Am. J. Clin. Nutr..

[68] Raikes A.C., Hernandez G.D., Mullins V.A., Wang Y., Lopez C., Killgore W.D.S. (2022). Effects of docosahexaenoic acid and eicosapentaoic acid supplementation on white matter integrity after repetitive sub-concussive head impacts during American football: exploratory neuroimaging findings from a pilot RCT. Front. Neurol..

[69] McLennan P.L., Pepe S. (2015). Weighing up fish and omega-3 PUFA advice with accurate, balanced scales: stringent controls and measures required for clinical trials. Heart Lung Circ.

[70] Schuchardt J.P., Tintle N., Westra J., Harris W.S. (2023). Estimation and predictors of the omega-3 index in the UK Biobank. Br. J. Nutr..

[71] Simopoulos A.P. (2007). Omega-3 fatty acids and athletics, Curr. Sports Med. Rep..

[72] Simopoulos A.P., DiNicolantonio J.J. (2016). The importance of a balanced ω-6 to ω-3 ratio in the prevention and management of obesity. Heart.

[73] DiNicolantonio J.J., O’Keefe J. (2021). The importance of maintaining a low omega-6/omega-3 ratio for reducing the risk of autoimmune diseases, asthma, and allergies, Mo. Med..

[74] Harris W.S., Varvel S.A., Pottala J.V., Warnick G.R., McConnell J.P. (2013). Comparative effects of an acute dose of fish oil on omega-3 fatty acid levels in red blood cells versus plasma: implications for clinical utility. J. Clin. Lipidol..

[75] Slee E.L., McLennan P.L., Owen A.J., Theiss M.L. (2010). Low dietary fish-oil threshold for myocardial membrane n-3 PUFA enrichment independent of n-6 PUFA intake in rats. J. Lipid Res..

[76] Macartney M.J., Peoples G.E., Treweek T.M., McLennan P.L. (2019). Docosahexaenoic acid varies in rat skeletal muscle membranes according to fibre type and provision of dietary fish oil. Prostaglandins Leukot. Essent. Fatty Acids.

[77] Sinclair A.J. (2019). Docosahexaenoic acid and the brain – what is its role? Asia Pac. J. Clin. Nutr..

